# Intraspecific Correlations of Basal and Maximal Metabolic Rates in Birds and the Aerobic Capacity Model for the Evolution of Endothermy

**DOI:** 10.1371/journal.pone.0034271

**Published:** 2012-03-27

**Authors:** David L. Swanson, Nathan E. Thomas, Eric T. Liknes, Sheldon J. Cooper

**Affiliations:** 1 Department of Biology, University of South Dakota, Vermillion, South Dakota, United States of America; 2 Department of Biology, Northern State University, Aberdeen, South Dakota, United States of America; 3 Department of Biology and Microbiology, University of Wisconsin-Oshkosh, Oshkosh, Wisconsin, United States of America; Roehampton University, United Kingdom

## Abstract

The underlying assumption of the aerobic capacity model for the evolution of endothermy is that basal (BMR) and maximal aerobic metabolic rates are phenotypically linked. However, because BMR is largely a function of central organs whereas maximal metabolic output is largely a function of skeletal muscles, the mechanistic underpinnings for their linkage are not obvious. Interspecific studies in birds generally support a phenotypic correlation between BMR and maximal metabolic output. If the aerobic capacity model is valid, these phenotypic correlations should also extend to intraspecific comparisons. We measured BMR, M_sum_ (maximum thermoregulatory metabolic rate) and MMR (maximum exercise metabolic rate in a hop-flutter chamber) in winter for dark-eyed juncos (*Junco hyemalis*), American goldfinches (*Carduelis tristis;* M_sum_ and MMR only), and black-capped chickadees (*Poecile atricapillus*; BMR and M_sum_ only) and examined correlations among these variables. We also measured BMR and M_sum_ in individual house sparrows (*Passer domesticus*) in both summer, winter and spring. For both raw metabolic rates and residuals from allometric regressions, BMR was not significantly correlated with either M_sum_ or MMR in juncos. Moreover, no significant correlation between M_sum_ and MMR or their mass-independent residuals occurred for juncos or goldfinches. Raw BMR and M_sum_ were significantly positively correlated for black-capped chickadees and house sparrows, but mass-independent residuals of BMR and M_sum_ were not. These data suggest that central organ and exercise organ metabolic levels are not inextricably linked and that muscular capacities for exercise and shivering do not necessarily vary in tandem in individual birds. Why intraspecific and interspecific avian studies show differing results and the significance of these differences to the aerobic capacity model are unknown, and resolution of these questions will require additional studies of potential mechanistic links between minimal and maximal metabolic output.

## Introduction

The assumption of a positive phenotypic correlation between basal metabolic rate (BMR, minimum maintenance metabolic rate) and maximum metabolic rates is the basis for the aerobic capacity model of the evolution of endothermy in birds and mammals [1]. However, different physiological factors are primarily responsible for BMR and maximum metabolic capacities. BMR is primarily a function of central organs, whereas maximal metabolic output is primarily a function of skeletal muscles [2], [3]. Maximum metabolic output in endotherms is determined either as exercise-induced maximum metabolic rate (hereafter, MMR) or as thermogenic maximum metabolic rates during cold exposure (hereafter, Summit Metabolic Rate or M_sum_). Whereas both MMR and M_sum_ represent maximum metabolic outputs (from exercise and shivering, respectively), MMR generally exceeds M_sum_ in endotherms, with factorial aerobic scopes (Maximum metabolic output/BMR) in birds generally ranging from 8–14 for MMR and from 4–8 for M_sum_ [4]. Similarly, Wiersma et al. [5] showed that MMR (measured during exercise in a hop-flutter wheel) exceeded M_sum_ for tropical birds by an average of 47%, although both scaled similarly with body mass.

BMR in birds is related to latitude and climate, increasing away from the tropics and in colder climates and elevated in temperate-zone birds relative to tropical birds [6], [7], [8], [9]. Climate also influences M_sum_ in birds, with birds wintering in colder climates having higher baseline M_sum_ than birds wintering in warmer climates [10], [11]. In addition, both BMR and M_sum_ typically vary seasonally in response to changing energy demands, generally increasing in winter relative to summer for birds in cold climates [4], [12], [13] and increasing during migration relative to non-migratory periods [14], [15], [16], [17]. Such coupled variation in response to seasonally changing energy demands (but see [18], [19]) also suggests a phenotypic correlation between minimum and maximum metabolic output in birds. Moreover, interspecific studies examining correlations between BMR and maximum metabolic output (both MMR and M_sum_) in birds and mammals generally show positive phenotypic correlations [5], [20], [21], [22], but this is not always the case. For example, Wiersma et al. [5] documented significant positive phenotypic correlations between BMR and MMR, but not between BMR and M_sum_. Nevertheless, the majority of interspecific studies on birds do support a correlation between minimum and maximum metabolic output, which is consistent with the assumptions of the aerobic capacity model for the evolution of endothermy [5], [20], [22].

If the aerobic capacity model assumption of a mechanistic linkage between minimum and maximum metabolic output is valid, such a correlation should be demonstrable for both inter- and intraspecific comparisons. Intraspecific correlations between minimum and maximum metabolic rates in birds and mammals have been little studied. Chappell and Bachman [23] examined BMR, M_sum_ and MMR in Belding's ground squirrels (*Spermophilus beldingi*) and found that mass-independent residuals of BMR and MMR were significantly positively correlated, but that residuals of BMR and M_sum_ were not, although contributions to M_sum_ from non-shivering thermogenesis via brown fat, in addition to muscular thermogenesis, complicate interpretation of the relationship between BMR and M_sum_ in mammals. BMR and M_sum_ were significantly positively correlated in red knots (*Calidris canutus*), but their mass-independent residuals were not, indicating that the correlation between BMR and M_sum_ was driven by variation in body mass [24]. However, Lewden et al. [25] found that both raw and mass-independent values for BMR and M_sum_ were significantly positively correlated in winter black-capped chickadees (*Poecile atricapillus*). Thus, some evidence for intraspecific phenotypic correlations between BMR and exercise-induced maximum metabolic output exists, but the few studies to date are equivocal in their support for a correlation between mass-independent BMR and maximum metabolic output for thermogenesis.

Because shivering thermogenesis in birds relies heavily on the flight muscles [26], [27], [28], which are also used to support exercise, a correlation between M_sum_ and exercise-induced MMR might be expected. In addition, mechanisms underlying phenotypically flexible responses of metabolic output to variation in energy demand are often similar between migration and cold acclimation/acclimatization, including such changes as flight muscle hypertrophy and elevated cellular aerobic capacity [4], [29], further suggesting a phenotypic correlation between MMR and M_sum_. Indeed, migratory disposition in red knots produced thermogenic side effects in the absence of temperature differences [17] and M_sum_ is elevated in migratory passerines during spring migration periods, consistent with selection for endurance flight producing increases in M_sum_ as a by-product [14], [16]. These data support the existence of a positive phenotypic correlation between M_sum_ and MMR in birds. To our knowledge, only one study has examined such a correlation directly, but Wiersma et al. [5] found that mass-independent residuals of MMR and M_sum_ were not correlated in an interspecific comparative study of tropical birds.

Thus, current data are generally supportive of phenotypic correlations between minimum and maximum metabolic output in birds, but exceptions to this generalization exist and very few studies have directly addressed whether such correlations occur on an intraspecific basis. Current data are equivocal with regard to phenotypic correlations between M_sum_ and MMR in birds, and to date no intraspecific studies have directly addressed this question. Our objective in the current study was to test for intraspecific correlations among BMR, MMR and M_sum_ in several species of passerine birds and we hypothesize that BMR, MMR, and M_sum_ are interrelated. More specifically, we predict that positive correlations will exist between BMR and M_sum_ and BMR and MMR and that M_sum_ and MMR will also be positively correlated.

## Materials and Methods

### Study Species and Experimental Design

Previous studies have tested for phenotypic correlations between BMR and maximal metabolic output in birds using both exercise (MMR) and thermogenic (M_sum_) maximum metabolic rates, and we used both approaches in this study. We measured all three metabolic variables (BMR, MMR and M_sum_) for individual dark-eyed juncos (*Junco hyemalis*). We did not measure all three metabolic variables on other study species. For American goldfinches (*Carduelis tristis*), because of time and equipment constraints (insufficient metabolism systems to measure BMR for both juncos and goldfinches concurrently), we measured only MMR and M_sum_ to test for correlations between these variables. For black-capped chickadees and house sparrows (*Passer domesticus*), we incorporated both published [21], [30] and unpublished data from studies where our objective was to examine seasonal or within-season variation in BMR and M_sum_, so we only measured these variables, but because both were measured on the same individual birds, we incorporated these data into the current study. All the study species show elevated BMR and M_sum_ in winter compared to summer [30], [31], [32], [33], [34], [35]. Juncos and chickadees also demonstrate negative relationships between metabolic rates and winter temperature [36], so winter represents a period of high, but variable, metabolic rates during a season where thermogenic capacity is at its annual zenith. Thus, winter should likely be the period during the annual cycle when phenotypic correlations between minimum and maximum (at least for M_sum_) metabolic output should be most likely to be detected. For additional metabolic comparisons, we included house sparrows sampled from three seasons to further increase variation in metabolic rates.

We collected dark-eyed juncos (n = 36), American goldfinches (n = 20) and black-capped chickadees (n = 13) in winter (December-February) at woodland sites near Vermillion, Clay County, South Dakota (approximately 43°N, 97°W). We used data from house sparrows collected both near Oshkosh, Winnebago County, Wisconsin (approximately 44°N, 89°W) and near Vermillion, South Dakota, from winter (December-early March), spring (April) and summer (late May-August). Data from individual Wisconsin birds include data from Arens and Cooper [30] and from spring South Dakota birds include data from Dutenhoffer and Swanson [21]. The sample sizes for the different seasons and study sites for the house sparrow data were: Wisconsin summer (n = 13); South Dakota spring (n = 7); Wisconsin winter (n = 11) and South Dakota winter (n = 8). For these studies, we transported birds from our study sites to the laboratory and completed all metabolic measurements on the day of capture to avoid potential effects of captivity on metabolic rates. We captured birds under valid federal and state scientific collecting permits and all procedures were approved by Institutional Animal Care and Use Committees and conformed to the Ornithological Council's *Guidelines for the Use of Wild Birds in Research*.

### Metabolic Measurements

We measured metabolic rates using open-circuit respirometry as described in Swanson et al. [37] for South Dakota birds and Arens and Cooper [30] for Wisconsin birds. We followed a standardized sequence for metabolic tests, with MMR measured first, followed by a rest period of at least two hours before M_sum_ measurement, which, in turn, was followed by a rest period of at least 5 hours before BMR measurement. For birds where only two of these three metabolic measurements were completed, we followed the same sequence with the omission of one of the metabolic measures. The respirometry system consisted of 1.8-L paint cans with the inside painted flat black (South Dakota birds [37]) or 1-L glass metabolic chambers (Wisconsin birds [30]). We controlled temperature within metabolic chambers to ±0.2°C by immersing chambers into a bath of water and ethylene glycol (South Dakota birds) or by placing chambers in a Hotpack incubator (Model 352602; Wisconsin birds). We maintained flow rates of dry, CO_2_-free air at 280–300 ml min^−1^ (South Dakota birds) or 488–520 ml min^−1^ (Wisconsin birds) for BMR and at 1,730–1,760 ml min^−1^ for MMR by either a Cole-Parmer Precision Rotameter (Model FM082–03ST; South Dakota birds) or an Omega (Model FMA-A2048) Mass Flow Controller (Wisconsin birds). We maintained flow rates of dry, CO_2_-free, helox between 1,000 and 1,150 ml min^−1^ with Cole-Parmer Precision Rotameters. We calibrated rotameters to ±1% accuracy for both air and helox with a soap bubble meter. We measured fractional concentrations of oxygen in excurrent air with Ametek S-3A (South Dakota birds) or Sable Systems FC-1B (Wisconsin birds) oxygen analyzers at 1 or 5 sec intervals and collected data with Datacan 5.0 software. We calibrated oxygen analyzers daily prior to measurements with ambient air. We analyzed metabolic data with Expedata 2.0 (Sable Systems, Henderson, NV) or Warthog Systems LabAnalyst (Riverside, CA) software after correcting to STPD.

We conducted BMR measurements at night (at least one hour after civil twilight) on birds fasted for at least four hours prior to metabolic measurements and at 30°C, which is within the thermoneutral zone of all study species [30], [33], [34], [38]. We allowed birds a 1-h equilibration period within the metabolic chamber before we initiated metabolic measurements. All birds tested showed low, stable metabolic rates, without metabolic variation suggesting activity, after the 1-h equilibration period. BMR measurements continued for 30 min (Wisconsin birds) or 1 h (South Dakota birds) following the equilibration period. In experiments on a subset of the Wisconsin sparrows, we found that metabolic rates recorded for the first 30-min after the 1-h equilibration period were consistent with metabolic rates recorded over the entire night.

We elicited M_sum_ using a sliding cold exposure protocol [37] with a 79% helium/21% oxygen gas mixture (helox). Helox increases heat loss without impairment of metabolic function so that maximal thermogenic metabolic rates can be obtained at relatively modest temperatures [30], [39], [40]. For the sliding cold exposure protocol, we first flushed the metabolic chamber with helox for 5 min prior to initiation of cold exposure to replace chamber air with helox. After this period, we initiated the cold exposure by placing the metabolic chamber into the anti-freeze bath or incubator. We continued the sliding cold exposure treatment until we detected a steady decline in oxygen consumption over several minutes, indicative of hypothermia. At this time we removed birds from the metabolic chamber and recorded body temperature (T_b_) with a thermocouple thermometer. We considered body temperatures of ≤36°C as hypothermic and all birds were hypothermic at the end of cold exposure trials, which validated that M_sum_ had been attained.

We used a hop-flutter chamber [5], [41], [42] to generate exercise-induced MMR. Our hop-flutter chamber was designed from a 30-cm diameter ×14-cm width piece of PVC pipe with acrylic side panels affixed to produce an air-tight seal. Incurrent and excurrent air passed through air-tight rotating steel fittings with attached diffusers to provide mixing of air in the wheel. We attached the chamber to a variable speed motor to control rotation speed and placed three ping-pong balls in the chamber to help motivate the bird to exercise as the wheel was turning [41]. We introduced birds into the chamber through a port with a removable air-tight cap. Prior to MMR measurements, we allowed a 5-min equilibration period, during which the chamber was covered by a sheet to calm the bird, before we initiated chamber rotation. After placing the bird in the chamber, we initiated chamber rotation at the lowest speed on the motor for 3 min and increased the rotation speed every 3 min thereafter until the oxygen consumption began to decrease and the bird showed reluctance to exercise. During the MMR protocol, birds typically hopped and engaged in short fluttering flights to maintain their position in the rotating chamber. At the termination of the MMR protocol, birds invariably showed signs of exhaustion (e.g., resting on their breast on the chamber floor and panting heavily), suggesting that maximum aerobic activity during the hop-flutter exercise had been attained.

We used steady-state equations [43] for calculating oxygen consumption for BMR and for M_sum_ and MMR we calculated instantaneous rates of oxygen consumption according to Bartholomew et al. [44]. For BMR measurements, we considered the lowest 10-min running mean over the test period as BMR. For M_sum_ measurements we considered the highest 5-min (South Dakota birds) or 10-min (Wisconsin birds) running mean over the test period as M_sum_ [5], [30]. We used the maximum 5-min running mean over the test period as MMR [5], [11].

### Statistical Analyses

We used least squares linear regression to analyze relationships between all metabolic variables and body mass and among BMR, M_sum_ and MMR. Both body mass (M_b_) and metabolic rate data were log_10_-transformed prior to regression analyses of allometric relationships. To remove the effects of M_b_ from analyses of relationships among metabolic variables, we calculated residuals from allometric regressions for BMR, M_sum_ and MMR and used linear regression of residuals. These residual analyses test whether individual birds with high or low values for one metabolic variable at a given M_b_ also have similarly high or low values at a given M_b_ for other metabolic variables. We compared M_b_, BMR and M_sum_ among house sparrows from different seasons and locations by one-way ANOVA, with Fisher's LSD test to identify differing means. We report data as means ± SD, unless otherwise noted. Statistical significance for all analyses was accepted at *P*≤0.05.

## Results

Mean BMR for dark-eyed juncos in this study (n = 23) was 1.241±0.123 ml O_2_ min^−1^ (mean M_b_ = 19.0±1.3 g). Mean M_sum_ (n = 33) and MMR (n = 36) for dark-eyed juncos were 7.581±0.825 ml O_2_ min^−1^ (mean M_b_ = 20.1±1.1 g) and 9.654±1.756 ml O_2_ min^−1^ (mean M_b_ = 20.8±1.3 g), respectively. Factorial scope for dark-eyed juncos for M_sum_ (M_sum_/BMR) was 6.11 and for MMR (MMR/BMR) was 7.78. MMR exceeded M_sum_ in juncos by 27%.

**Figure 1:**
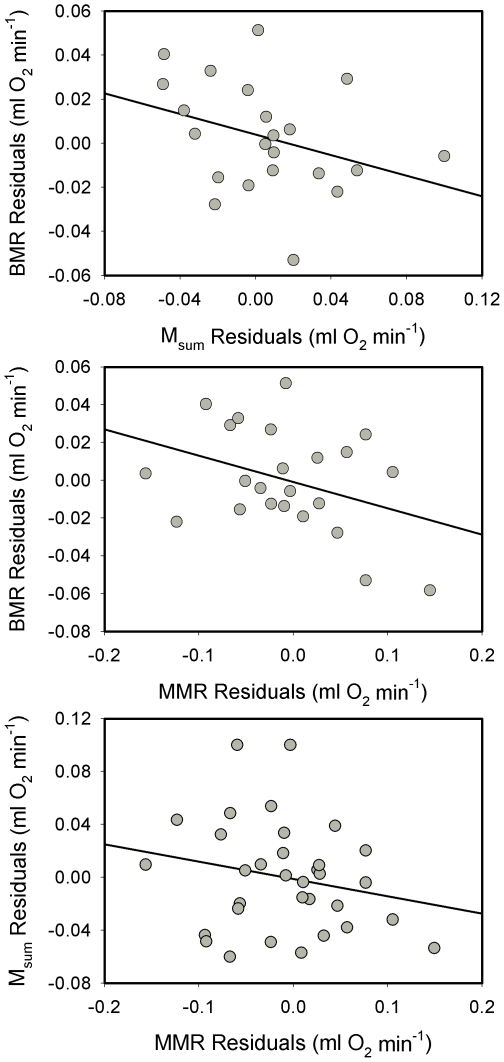
Correlations between mass-independent residuals of minimum and maximum metabolic rates for dark-eyed juncos . No significant correlations occurred for any of the comparisons. Statistics for the correlations were: BMR vs. M_sum_ (*R^2^* = 0.115, *P* = 0.123); BMR vs. MMR (*R^2^* = 0.134, *P* = 0.086); and M_sum_ vs. MMR (*R^2^* = 0.044, *P* = 0.241).

None of the correlations between raw BMR, M_sum_ or MMR were significant for dark-eyed juncos. Statistics for these correlations were: BMR vs. M_sum_, *R^2^* = 0.053, *P* = 0.301; BMR vs. MMR, *R^2^* = 0.115, *P* = 0.113; and M_sum_ vs. MMR, *R^2^* = 0.009, *P* = 0.603. Allometric regressions of log M_b_ vs. log metabolic rates for dark-eyed juncos were significant for BMR and M_sum_, but not for MMR (Table 1). Similar to raw metabolic rates, mass-independent residuals from allometric equations yielded no significant correlations among the different metabolic variables for dark-eyed juncos (Fig. 1).

**Table 1:** Allometric least squares regression equations for log metabolic rates (ml O_2_ min^−1^) against log body mass (Mb, g) for the four study species.

Species/Variable (n)	Intercept (± SE)	Slope (± SE)	*R^2^*	*P*
Dark-eyed junco				
BMR (23)	−1.425 (0.251)	1.186 (0.196)	0.635	<0.001
M_sum_ (30)	−0.173 (0.394)	0.806 (0.302)	0.187	0.012
MMR (33)	0.338 (0.624)	0.486 (0.473)	0.030	0.312
American goldfinch				
M_sum_ (20)	0.031 (0.486)	0.609 (0.427)	0.102	0.171
MMR (20)	−0.847 (0.926)	1.467 (0.819)	0.151	0.090
Black-capped chickadee				
BMR (13)	−1.258 (0.404)	1.144 (0.364)	0.473	0.009
M_sum_ (13)	−0.946 (0.480)	1.560 (0.428)	0.548	0.004
House sparrow				
BMR (39)	−1.554 (0.778)	1.167 (0.549)	0.109	0.040
M_sum_ (39)	−0.614 (0.670)	1.112 (0.467)	0.133	0.023

Sample sizes (n) for the different metabolic measurements are provided in parenthesis.

**Figure 2:**
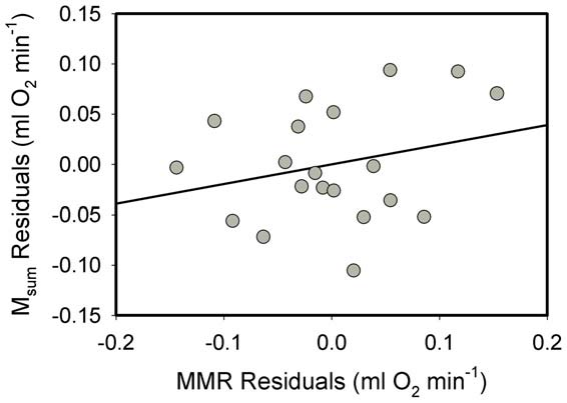
Correlation between mass-independent residuals of thermogenic (M_sum_) and exercise (MMR) metabolic rates for American goldfinches. The correlation was not significant (*R^2^* = 0.065, *P* = 0.279).

Mean metabolic rates (n = 20) for American goldfinches were 5.346±0.740 ml O_2_ min^−1^ (mean M_b_ = 13.8±1.0 g) for M_sum_ and 6.582±1.260 ml O_2_ min^−1^ (mean M_b_ = 13.5±0.7 g) for MMR. MMR exceeded M_sum_ in goldfinches by 23%. Similar to data for dark-eyed juncos, raw M_sum_ and MMR were not significantly correlated in goldfinches (*R^2^* = 0.115, *P* = 0.143). Moreover, allometric regressions for both log M_sum_ and log MMR against log M_b_ were not significant, although the regression for MMR approached significance (Table 1). Mass-independent residuals from allometric equations of M_sum_ and MMR were also not significantly correlated for goldfinches (Fig. 2).

**Figure 3:**
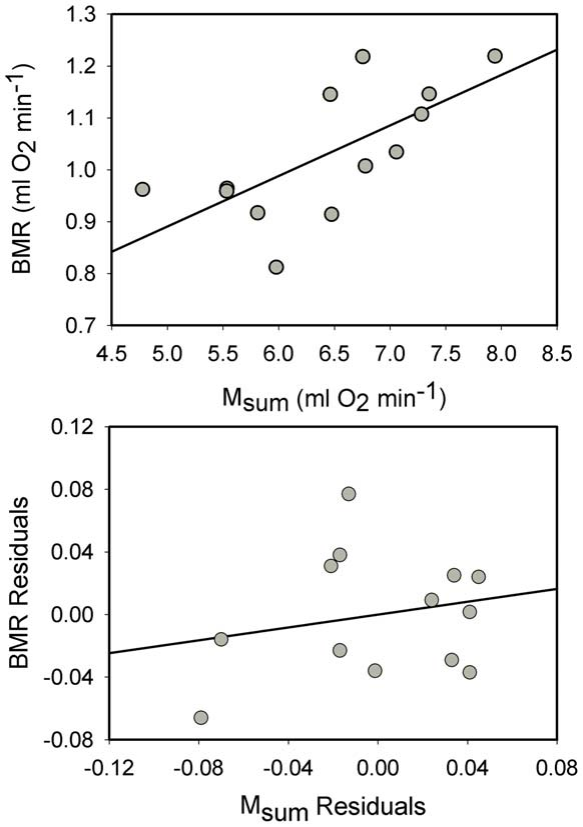
Correlations between BMR and M_sum_ for black-capped chickadees . Raw BMR and M_sum_ (upper panel) were significantly positively correlated, but mass-independent residuals (lower panel) were not (*R^2^* = 0.048, *P* = 0.470), indicating that the correlation between raw metabolic values was driven by body mass.

Mean metabolic rates for black-capped chickadees (n = 13) were 1.031±0.127 ml O_2_ min^−1^ (mean M_b_ = 12.9±0.9 g) for BMR and 6.442±0.886 ml O_2_ min^−1^ (mean M_b_ = 13.3±0.8 g) for M_sum_. Factorial scope for M_sum_ was 6.25. Raw BMR and M_sum_ were significantly positively correlated, with the least squares regression equation:
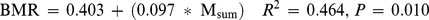
Allometric regressions of log M_b_ vs. log metabolic rates for black-capped chickadees were significant for both BMR and M_sum_ (Table 1). In contrast to raw metabolic rates, mass-independent residuals from allometric equations yielded no significant correlation between BMR and M_sum_ for black-capped chickadees (Fig. 3), indicating that the relationship between BMR and M_sum_ is driven by variation in body mass among individual birds.

**Figure 4:**
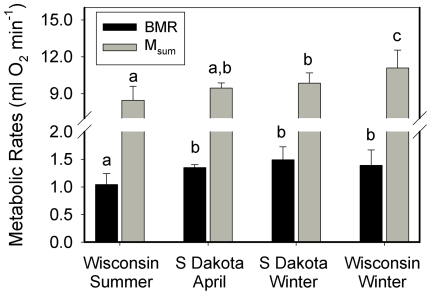
Seasonal and geographic variation in metabolic rates for house sparrows . Metabolic values with the same superscript do not statistically differ from each other. BMR and M_sum_ were both lowest in summer birds and elevated at other times of the year. M_sum_ for South Dakota (S Dakota) birds in April was nearly significantly greater than that for summer Wisconsin birds (P = 0.062).

Mean M_b_ did not differ significantly for house sparrows among seasons or locations and averaged 27.2±1.4 g (n = 39). Mean BMR, however, was significantly lower for summer birds than for birds from other seasons (Fig. 4). Mean M_sum_ also differed significantly among seasons and locations (Fig. 4), with M_sum_ highest in winter birds from Wisconsin and lowest in summer birds. M_sum_ of winter and April sparrows from South Dakota did not differ significantly from each other, but were significantly lower than winter birds from Wisconsin and significantly (or nearly significantly) higher than summer birds from Wisconsin (Fig. 4). Factorial scope for M_sum_ ranged from 6.6 for South Dakota winter birds to 8.1 for Wisconsin summer birds.

**Figure 5:**
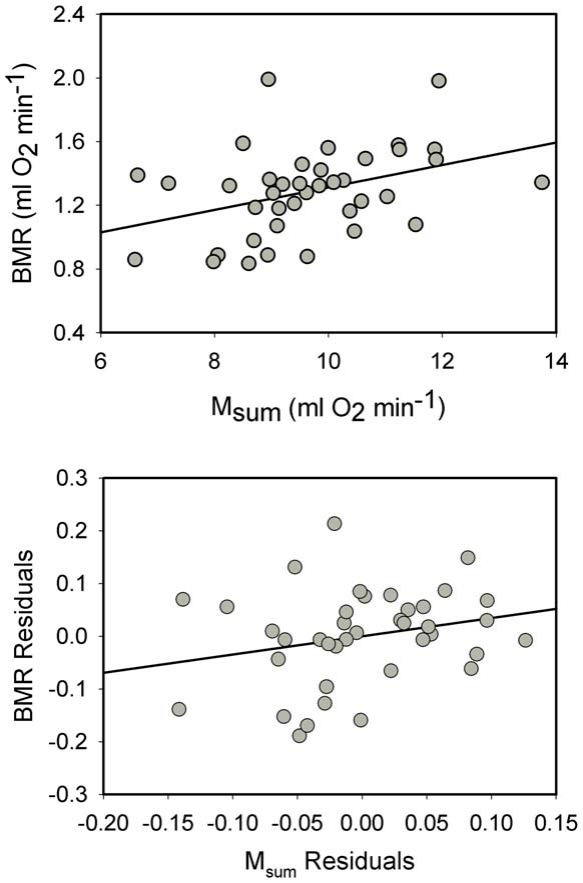
Correlations between BMR vs. M_sum_ for house sparrows . Raw BMR and M_sum_ (upper panel) were significantly positively correlated, but mass-independent residuals (lower panel) were not (*R^2^* = 0.060, *P* = 0.134), indicating that the correlation between raw metabolic values was driven by body mass.

Raw BMR and M_sum_ were significantly positively correlated for house sparrows, and the relationship was described by the least squares regression equation:
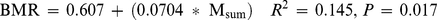
Allometric equations for log BMR and log M_sum_ against log M_b_ were significant for both BMR and M_sum_ (Table 1). Similar to American goldfinches, mass-independent residuals for log BMR and log M_sum_ for house sparrows were not significantly correlated (Fig. 5).

## Discussion

In general, the data from this study provide little evidence supporting the assumption of the aerobic capacity model for the evolution of endothermy (i.e., positive phenotypic correlations between minimum and maximum metabolic outputs) within bird species. Neither MMR nor M_sum_ were significantly correlated with BMR for dark-eyed juncos, either for raw metabolic values or for mass-independent residuals. Raw values for BMR and M_sum_ were positively correlated for both black-capped chickadees and house sparrows, but mass-independent residuals were not, which indicates that the correlation of raw values in these two species was driven by variation in body mass. The absence of significant intraspecific phenotypic correlations of mass-independent minimum and maximum metabolic outputs in this study contrasts with results from interspecific avian studies, which generally show positive correlations between mass-independent M_sum_ and BMR, at least for temperate-zone species ([21], [22], but see [5] for tropical species) or mass-independent MMR and BMR [5]. However, the data for chickadees and house sparrows are consistent with the intraspecific pattern documented by Vézina et al. [24] for red knots, where raw values for BMR and M_sum_ were significantly positively correlated, but mass-independent residuals were not. In contrast, both raw values and mass-independent residuals for BMR and M_sum_ were significantly positively correlated for winter black-capped chickadees from Quebec [25]. Why significant correlations of mass-independent BMR and M_sum_ occur for chickadees from Quebec but not from South Dakota is unknown. Because chickadees may seasonally alter both body composition and cellular aerobic capacity of muscles [45], [46], and variation in cellular aerobic capacity will contribute to changes in mass-independent metabolic rates more than variation in body composition, the differences between the two populations could conceivably result from differing contributions of adjustments in body composition and cellular aerobic capacity to winter acclimatization in the two populations.

Given the absence of intraspecific correlations between mass-independent BMR and maximal metabolic output in this study, it is reasonable to ask why interspecific and intraspecific correlations between minimum and maximum metabolic outputs might differ in birds. One factor potentially affecting the differential intra- and interspecific relationships is the total amount of variation in metabolic values. Interspecific comparisons include birds from a wider range of body sizes and phylogenetic affinities and thus show a much wider spread for the metabolic data than intraspecific comparisons. This larger variation in metabolic values could provide a greater level of resolution for detecting phenotypic correlations between metabolic values. In support of this idea, slopes for regressions of log metabolic rates against log M_b_ in birds show much greater variation for intraspecific studies than for interspecific studies, and slopes are often higher for intraspecific studies [13]. This suggests that the amount of variation in body mass can affect the scaling exponent, with wider ranges of M_b_ providing a better overall view of how metabolic rates vary with M_b_ across a broad phylogenetic sample within a particular taxon. Similarly, a greater total variation in metabolic rates, as provided by interspecific studies, could produce a better overall view of phenotypic correlations of minimum and maximum metabolic outputs.

The absence of intraspecific phenotypic correlations between mass-independent minimum and maximum metabolic output for birds in this study is contrary to predictions from the aerobic capacity model for the evolution of endothermy, which requires a phenotypic link between basal and maximum metabolic rates. Thus, these data suggest that metabolic intensities of central organs (which largely determine basal metabolic rates) and exercise organs (which largely determine maximal capacities for exercise and shivering) are not inextricably linked in individual birds. However, chickadees and house sparrows did show positive phenotypic correlations between raw values for BMR and M_sum_, although juncos did not. An argument could be made that raw values for metabolic rates are the more appropriate metric for examining intraspecific correlations between minimum and maximum metabolic output because a prominent mechanism for phenotypic flexibility of metabolic rates in birds is to adjust body composition (i.e., the size of the organs rather than their metabolic intensity [4], [47]). Such an argument has been made previously for comparisons of seasonal variation in metabolic rates in birds [31], [48]. Mass-independent metabolic rates assume a constant contribution of mass to metabolic rates, but because tissues and organs differ in metabolic intensity, increases in the masses of metabolically active tissues or organs will contribute disproportionately to increases in metabolic rates. Similarly, because fat is relatively inert metabolically, variation in fat mass among individuals could also confound detection of correlations between mass-independent measures of minimum and maximum metabolic output. In such cases mass-independent metabolic rates may not be the most effective metric for examining metabolic correlations. Indeed, differences in body composition may also underlie large-scale ecological differences in metabolic rates among species, such as the differences in basal and maximal metabolic rates between temperate and tropical bird species [5]. If adjustments in sizes of metabolically important organs are important drivers of intraspecific metabolic variation, then the positive intraspecific phenotypic correlations for raw metabolic output, but the absence of mass-independent correlations, as documented for chickadees and house sparrows in this study, may still offer general support for the aerobic capacity model. In any event, more research directed at understanding mechanisms of phenotypic linkage (or the lack thereof) between minimum and maximum metabolic rates in birds and other vertebrates are needed to help resolve these questions.

**Figure 6:**
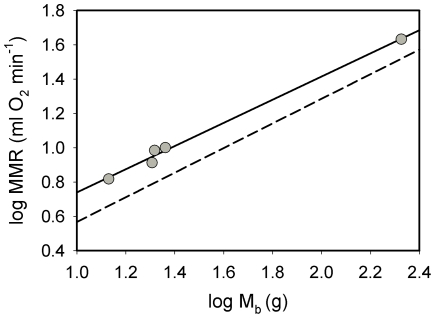
Allometric relationships for MMR in temperate-zone birds . Least squares allometric regression for log MMR (measured in a hop-flutter wheel) against log M_b_ for five species of temperate-zone birds for which MMR has been recorded (solid line). For comparison, the allometric regression equation for MMR for tropical birds from [5] is included as the dashed line. MMR values for other temperate-zone bird species include satin bowerbird [49], red-eyed vireo [42] and house sparrow [41].

Factorial aerobic scopes for thermogenesis (M_sum_/BMR) in this study ranged from 6.1 for juncos to 8.1 for summer house sparrows from Wisconsin. These scopes are consistent with factorial scopes for thermogenesis for other birds, which generally range from 4–8 [4], with a maximum value of 9.0 from a previous study of summer-acclimatized house sparrows from Wisconsin [30]. Factorial aerobic scope for exercise in the hop-flutter wheel (MMR/BMR) was 7.8 for dark-eyed juncos in this study, a value lower than those for other temperate-zone bird species, which include 10.4 for red-eyed vireo [42], 10.6 for house sparrows [41] and 11.2 for satin bowerbirds (measured from allometrically predicted BMR [49]). Using the BMR value for winter-acclimatized American goldfinches from South Dakota from Liknes et al. [34] of 1.04 ml O_2_ min^−1^, gives and estimated hop-flutter exercise factorial aerobic scope for goldfinches of 6.3, which is also lower than that for the other temperate-zone species. However, the lower exercise factorial aerobic scope for juncos and goldfinches in this study is not due to lower MMR, as the MMR data for these two species fit in well with those for the other temperate-zone species (Fig. 6, *R^2^* for regression of log MMR on log M_b_ = 0.994). The lower scopes likely result from a relatively higher BMR, which is not surprising given that our measurements were conducted in winter birds from cold climates, whereas measurements from the other temperate-zone species were not, and BMR is typically elevated in winter for birds from cold climates [4], [12], [13]. This brings up the interesting possibility that exercise factorial aerobic scopes may vary seasonally in birds from cold climates, but confirmation of this possibility will require further research.

The exercise factorial aerobic scopes for goldfinches and juncos in our study actually more closely approximate exercise factorial aerobic scopes for tropical species, which average 6.44. Thus, exercise factorial aerobic scopes may not greatly differ between temperate-zone and tropical species, as Wiersma et al. [5] tentatively suggest. Nevertheless, our MMR data do support the contention of Wiersma et al. [5] that temperate-zone birds have higher MMR than tropical birds, which is consistent with the general pattern of a slower pace of life in tropical birds [8]. MMR for goldfinches and juncos exceeded allometric predictions for tropical birds [5] by 61.3 and 70.5%, respectively.

Exercise MMR typically exceeds thermogenic M_sum_ in birds, with maximum factorial aerobic scopes during flight or running exceeding 20 [50], [51], [52], [53]. Several potential reasons exist for higher MMR than M_sum_, with the most plausible being differences in the mass of muscle recruited for exercise and shivering, differences in body temperature during exercise and cold exposure, and differences in blood flow to the working muscles between isotonic exercise and isometric shivering [4], [54]. For tropical bird species in which MMR during hop-flutter wheel exercise and M_sum_ were both measured, MMR exceeded M_sum_ by an average of 47% [5]. For juncos and goldfinches in this study, MMR exceeded M_sum_ by 27% and 23%, respectively. These values are lower than the values for tropical birds, which suggests that M_sum_ may comprise a greater fraction of MMR in temperate-zone birds than in tropical birds. A higher relative M_sum_ in temperate-zone birds is consistent with general patterns of climatic effects on M_sum_, with birds from cold climates having elevated M_sum_, even in summer when temperatures are not cold [10], [11]. M_sum_ is further elevated in winter, often by 20–50%, for birds inhabiting cold winter climates [4], which would potentially serve to further elevate the relative fraction of MMR comprised by M_sum_, and both juncos and goldfinches in this study were from the cold winter climates of South Dakota. This might also help explain the relatively smaller difference between MMR and M_sum_ in this study compared to tropical species [5].

Because both exercise and shivering represent forms of muscular activity and many of the mechanistic bases supporting elevated capacities for endurance exercise and prolonged shivering are similar in birds [4], [29], [54], it might be expected that MMR and M_sum_ would be phenotypically correlated. Supporting this notion are elevated M_sum_ during migration in passerine birds [14], [16] and elevated M_sum_ during migratory disposition in red knots acclimated to standard temperature exposure treatments [17]. However, M_sum_ and MMR were not significantly correlated for juncos and goldfinches in this study, for either raw metabolic rates or mass-independent residuals. The absence of such a correlation was also documented for tropical birds [5]. Thus, despite similar mechanistic underpinnings, muscular capacities for exercise and shivering do not appear to vary in tandem for bird species measured to date.

Our data for house sparrows allow some seasonal and geographic comparisons of metabolic rates in this species. In general, values for BMR and M_sum_ recorded for house sparrows in this study (Fig. 4) were within the range of previously recorded values, which range from 0.84 to 1.82 ml O_2_ min^−1^ for BMR [21], [30], [55], [56], [57] and from 7.0 to 10.9 ml O_2_ min^−1^ for M_sum_ [21], [30], [35], [58], although mean M_sum_ for winter sparrows from Wisconsin in this study (11.1 ml O_2_ min^−1^) slightly exceeded previous values for M_sum_ for this species. BMR in house sparrows in this study showed typical patterns of seasonal variation, with summer BMR lower than that for April and winter for birds from both South Dakota and Wisconsin. No geographic variation in winter BMR was evident in this study as winter BMR was not different between Wisconsin and South Dakota birds. Season and location both influenced M_sum_ for house sparrows in this study, with winter birds having higher M_sum_ than summer birds and M_sum_ for April birds being intermediate, and winter birds from Wisconsin having higher M_sum_ than winter birds from South Dakota. This pattern of geographic variation in M_sum_ is opposite to that from black-capped chickadees from Ohio, Wisconsin and South Dakota, where chickadees from South Dakota had higher winter M_sum_ than chickadees from either Wisconsin or Ohio [59]. Olson et al. [59] suggested that geographic differences in M_sum_ in chickadees might be related to woodland area, which is typically smaller in South Dakota than in Wisconsin or Ohio, and the attendant increases in energetic costs from higher convective heat losses due to increased wind penetration into smaller woodland parcels. Because house sparrows are often associated with human habitation, such potential differences in convective heat loss between South Dakota and Wisconsin might be buffered by behavioral use of buildings or thick vegetation around houses or buildings, which could help explain why house sparrows and chickadees show different geographic patterns of M_sum_ variation. In addition, because small birds show among-winter variation in metabolic rates related to the severity of the winter weather, with higher metabolic rates during cold winters [36], and house sparrow data were generated from different years in South Dakota and Wisconsin, differences in proximate winter weather conditions between the two sites could help account for the higher M_sum_ for Wisconsin birds in this study. A final possibility for why chickadees and house sparrows show different geographic patterns in metabolic variation with climate is that geographic variation in metabolic rates is not always correlated with geographic variation climatic in small birds. For example, house finches from Colorado and Michigan had higher winter M_sum_ than birds from California, supporting the idea of a link between winter climate and M_sum_ [60], [61]. However, interpretation of this pattern is complicated by the absence of seasonal variation in M_sum_ for California and Colorado birds, despite the colder winter temperatures in Colorado, but winter increments of M_sum_ for Michigan birds. Dark-eyed juncos from South Dakota and Oregon provide another example of the imperfect fit between climate and metabolic rates, as these birds did not show significant variation in winter M_sum_, despite markedly colder winters in South Dakota [38]. These data suggest that other factors in addition to temperature might also impact metabolic performance, but identification of these factors and the nature of their influence on metabolic rates will require additional research.

In summary, we found little intraspecific support for a phenotypic correlation of minimum and maximum metabolic output in birds, independent of mass, in this study. The absence of such a correlation does not support the assumption of the aerobic capacity model for the evolution of endothermy, which requires a phenotypic linkage between minimum and maximum metabolic rates. Raw values for minimum and maximum metabolic rates, however, were often, although not always correlated, suggesting that their correlation is driven by variation in body mass. The implications of these findings for the aerobic capacity model for the evolution of endothermy will require additional studies addressing potential mechanistic links between minimum and maximum metabolic output in birds and other vertebrate groups.
